# Argasid Ticks of Palearctic Bats: Distribution, Host Selection, and Zoonotic Importance

**DOI:** 10.3389/fvets.2021.684737

**Published:** 2021-06-22

**Authors:** Attila D. Sándor, Andrei Daniel Mihalca, Cristian Domşa, Áron Péter, Sándor Hornok

**Affiliations:** ^1^Department of Parasitology and Parasitic Diseases, University of Agricultural Sciences and Veterinary Medicine, Cluj-Napoca, Romania; ^2^Department of Parasitology and Zoology, University of Veterinary Medicine, Budapest, Hungary

**Keywords:** Chiroptera, host-specificity, Ixodoidea, soft ticks, zoonotic diseases

## Abstract

The soft ticks (Ixodida: Argasidae) are ectoparasites of terrestrial vertebrates with a wide geographic distribution, occurring on all continents. These ticks are obligate blood-feeders, most of them show high degrees of host-specialization and several species in arid and tropical regions are important parasites of livestock and men. Species commonly occurring on domestic animals and man are generally well-known, with many studies focusing on their ecology, distribution or vectorial role. However, wildlife-specialist soft ticks are less studied. Nearly half of all soft tick species are bat specialists, with five species (*Carios vespertilionis, Chiropterargas boueti, Chiropterargas confusus, Reticulinasus salahi*, and *Secretargas transgariepinus*) occurring in the Western Palearctic. There is no comprehensive study on the distribution, hosts or pathogens in these soft ticks, although most species were shown to carry several viral, bacterial, or protozoan pathogens and also to occasionally infest humans. Based on a literature survey and 1,120 distinct georeferenced records, we present here the geographical range, host selection and vectorial potential for bat-specialist soft ticks occurring in the Western Palearctic (chiefly Europe, North Africa, and the Middle East). *Carios vespertilionis* shows the largest distribution range and was found on most host species, being ubiquitous wherever crevice-roosting bats occur. All the other species were located only in areas with Mediterranean climate, with *Ch. boueti, Chiropteraragas confusus*, and *R. salahi* are missing entirely from Europe. These three species have a host spectrum of bats roosting primarily in caves, while *S. transgariepinus* and *Ca. vespertilionis* is feeding primarily on crevice-roosting bat species. All but one of these soft tick species are known to feed on humans and may be vectors of important disease agents (*Rickettsia* spp., *Borrelia* spp., *Bartonella* spp., *Ehrlichia* spp., *Babesia* spp., several nairo-, and flaviviruses). As several crevice-roosting bat species show a continuous adaptation to human-altered areas, with certain species becoming common city-dwellers in the Western Palearctic, the study of bat specialist soft ticks is also important from an epidemiologic point of view.

## Introduction

Ticks (Ixodoidea) are obligate blood-feeding arthropods, with a wide-spread occurrence and ~935 species known as parasites of terrestrial vertebrates (1, 2). The group has an ancient origin, with their first appearance suggested for the Cretaceous [65–146 mya, (3)], and widespread diversification and dispersal occurring during the Tertiary some 5 to 65 mya ago (4). Taxonomically, modern ticks are divided into three families (Argasidae, Ixodidae, and Nuttalliellidae) with the two most speciose being the hard ticks (Ixodidae) with 742 recognized species (2), followed by soft ticks (Argasidae) with 193 species, as listed in the last comprehensive checklist of this later group (1). The Nuttalliellidae consist of a single species (*Nuttalliella namaqua*), and is considered to be the most ancient among the three tick families, showing several intermediate characters specific for the other two (5).

Argasidae includes two subfamilies, Argasinae and Ornithodorinae, both with several genera, and subgenera, with differing numbers according to different authors (1, 6–10). They have a world-wide distribution, with most species being distributed in the tropics and dry regions of the globe (6). Argasid ticks show diverse adaptation to using their hosts. Most members of the family are characterized by a single, prolonged larval blood feeding and multiple, short blood feeding events of subsequent developmental stages on several host individuals, however other adaptations (e.g., no larval feeding or lack of blood-feeding in adults, etc.) were recorded in certain species (11). By doing so, these ticks are capable of taking up pathogens (viral, bacterial, or protozoan) and transfering them to other hosts, thus they have important vectorial role (6). Most of soft ticks inhabit holes and crevices and have access to hosts only occasionally, hence they developed extreme adaptations to prolonged fasting and short feeding bouts whenever hosts are available (12). Their vectorial capacity for several important zoonotic diseases is well-known, including human relapsing fever (its causative agent transmitted by *Ornithodoros* spp.), tick-borne relapsing fevers (caused by several *Borrelia* spp. transmitted mainly by *Ornithodoros* and *Argas* spp.) or African swine fever (vectored by *Ornithodoros moubata, Ornithodoros porcinus, Ornithodoros erraticus*, or *Ornithodoros savignyi*) causing severe economic losses (6, 13).

Soft ticks have a special relationship with bats (Mammalia: Chiroptera). Bats are widely distributed, show high species diversity (being the second largest order of mammals) and several adaptations, which make them ideal host candidates for tick parasitism (14). Their morphological adaptations for flight hinders the range of their behavioral responses to reduce tick burden (e.g., their highly specialized limbs are inadequate for proper grooming), most species are social, spending their resting periods in dense groups and they are highly attached to their specific roosting sites, of which most are either underground (caves) or crevices in rocks or trees—excellent hiding places for soft ticks (11). Thus, several soft tick species-groups evolved specific associations with bat hosts. For example, all the known 17 species of the Nearctic soft tick genus *Antricola* (and *Parantricola*) are exclusive parasites of bats (15), together with all species belonging to the subgenus *Carios, Chiropterargas, Nothoaspis*, and *Reticulinasus*, and several other species from the genera *Alectorobius* and *Ornithodoros* (Supplementary Material and references therein). While most of these soft tick species are tropical in their distribution, there are at least five species which regularly occur on bats in the Western Palearctic. These species are *Carios vespertilionis, Chiropterargas boueti, Chiropteraragas confusus, Reticulinasus salahi*, and *Secretargas transgariepinus*. All these parasitize bats mainly roosting either inside caves (*Ch. boueti, Chiropteraragas confusus*, and *R. salahi*) or crevices (*Ca*. *vespertilionis* and *S. transgariepinus*).

Our knowledge on the distribution and ecology of bat-specialist soft tick species is scanty, as most of the literature only lists occurrence records or describe specific case reports, without a systematic review on their range, status and importance. Here, we collated the published records on these five soft tick species in the Western Palearctic, looking for data on their geographical distribution, host-parasite relationships and vectorial importance and also raising awareness on future challenges posed by some of these species on human health. In the wake of recent climate change events and urbanization trends in bats' distribution, we also intended to look for the abiotic (climate linked) and biotic (host distribution linked) factors regulating the distribution of bat specialist soft ticks in the Western Palearctic.

## Materials and Methods

### Database Creation

Our methodology followed a three-step algorithm. First a database search was performed, using keywords as: “soft ticks,” “bats,” “Argasidae,” and “Western Palearctic,” “*Argas boueti*,” “*Argas confusus*,” “*Argas transgariepinus*,” “*Argas vespertilionis*,” and “*Ornithodoros salahi*” in the following databases: Web of Science, Zoological Record, Google Scholar, and Global Biodiversity Information Facility (www.gbif.org). Subsequently, copies of the original publications were obtained and the references cited in these works were traced. This process was repeated until no new references were found. In the third step we extracted each individual host-tick record from the references, noting the location, date, host and parasite species, development stage (for ticks) and pathogen (if) mentioned. These records were introduced into a database and individually georeferenced to create distribution maps.

### Distribution Maps

For the maps, we overlaid the different hosts' range with the presence data for each tick species. Each host range was set with transparency, so the more ranges overlap, the more intense the range color is—a proxy for multiple host species presence. For host ranges of main bat host species we used the freely available shape files from the website of the International Union for Conservation of Nature (IUCN) Red List (16). IUCN ranges were used previously primarily for conservation biology of bats (17) or other mammals (18), but also for establishing the relationships between bats, insect ectoparasites and their vectored pathogens (19). In the following step, we intersected the ranges with the contour of the Western Palearctic. Western Palearctic contour was delimited following the borders previously published (20, 21).

### Host-Parasite Relationships

Using the database we mapped each host-parasite relationship and delimited the primary/accidental hosts. For deciding primary/accidental hosts of any soft tick species we used an arbitrary rule. Any bat species which held more than 5.0% of any specific soft tick's record is considered a primary host of the respective tick species, while hosts with <5.0 % of all cumulative records of a particular tick are considered accidental hosts, following a system previously proposed for bat-bat fly associations (22, 23).

## Results

The complete database contains altogether 1,151 entries (4,856 individual ticks), collected from 899 hosts (4,378 ticks), together with a total of 65 cases of free ticks (involving 313 individuals), while collection circumstances were unknown for 156 cases (*n* = 165 ticks, only tick species and geographic location known). Altogether 44 bat species were recorded to host soft ticks, with most records noted for *Ca. vespertilionis* (Table 1). For a number of 16 cases the records mention only generic Chiroptera, while seven cases were assigned either to *Myotis* spp., *Pipistrellus* spp., or *Plecotus* spp. For 19 cases (1.9% of all records) the host is known, but it is not a bat species: 16 cases refer to humans, while one case each refer to a bird (*Picus viridis*), to a dog (*Canis familiaris*), while one to a rodent (*Allactaga euphratica*). Host species are listed in Tables 2, 3. *Carios vespertilionis* had the most diverse host spectrum, with altogether 42 different host species (6 primary and 36 secondary hosts), *Ch. boueti* had the most primary hosts (14), while *R. salahi* had a single primary host holding 87.7% of all records. Most ticks were recorded on crevice-dwelling bat species (76.6%), although for three species (*Ch. boueti, Chiropteraragas confusus*, and *R. salahi*) most primary bat hosts are cave-dwelling ones (24).

**Table 1:** Bat-specialist ticks recorded in the Western Palearctic.

	**Free stages**	**Collected from host**	**Total number of host species**	**Number of primary host species**	**Number of secondary hosts**	**Non-bat host species**	**Unknown/Undefined host**	**Total**
*Chiropterargas boueti*	2	16	14	14	0	1	1	19
*Chiropterargas confusus*	1	13	9	4	3	1	1	15
*Secretargas transgariepinus*	5	43	12	4	8	1	8	56
*Carios vespertilionis*	55	812	42	6	36	3	145	1,012
*Reticulinasus salahi*	2	15	4	1	2	1	1	18
TOTAL	65	899	44			3	156	1,120

*Number of records with known hosts, free stages, and host-types*.

**Table 2:** Primary and secondary bat host species of soft ticks (Argasidae) in the Western Palearctic.

**Tick species**	**Primary host species**	**Secondary host species**	**Non-bat hosts**
*Chiropterargas boueti*	*Asellia tridens, Nycteris thebaica, Otonycteris hemprichii, Pipistrellus kuhlii, Pipistrellus christii, Rhinolophus clivosus, Rhinolophus mehelyi, Rhinopoma cystops, Rhinopoma microphyllum, Rousettus aegyptiacus, Tadarida aegyptiaca, Tadarida teniotis, Taphozous nudiventris, Taphozous perforatus*	–	*Homo sapiens*
*Chiropterargas confusus*	*Asellia tridens, Nycteris thebaica, Otonycteris hemprichii, Pipistrellus kuhlii, Rhinolophus ferrumequinum, Rhinopoma cystops, Tadarida aegyptiaca, Taphozous nudiventris, Taphozous perforatus*	–	*Allactaga euphratica*
*Secretargas transgariepinus*	*Eptesicus serotinus, Eptesicus isabellinus, Plecotus austriacus, Hypsugo savii*	*Myotis emarginatus, Myotis myotis, Myotis mystacinus, Otonycteris hemprichii, Pipistrellus nathusii, Plecotus christii, Rhinolophus ferrumequinum, Rhinopoma cystops*	*Homo sapiens*
*Carios vespertilionis*	*Eptesicus serotinus, Myotis mystacinus, Nyctalus noctula, Pipistrellus kuhlii, Pipistrellus nathusii, Pipistrellus pipistrellus, Vespertilio murinus*	*Asellia tridens, Barbastella barbastellus, Eptesicus isabellinus, Eptesicus nilssoni, Hypsugo savii, Miniopterus pallidus, Miniopterus schreibersii, Myotis alcathoe, Myotis bechsteinii, Myotis blythii, Myotis brandtii, Myotis dasycneme, Myotis daubentonii, Myotis emarginatus, Myotis myotis, Myotis nattereri, Nyctalus lasiopterus, Nyctalus leisleri, Otonycteris hemprichii, Pipistrellus maderensis, Pipistrellus pygmaeus, Plecotus auritus, Plecotus austriacus, Plecotus christii, Plecotus gaisleri, Rhinolophus ferrumequinum, Rhinolophus mehelyi, Rhinopoma cystops, Rousettus aegyptiacus, Tadarida teniotis, Taphozous nudiventris, Rhinolophus blasii*	*Homo sapiens, Canis familiaris, Picus viridis*
*Reticulinasus salahi*	*Rousettus aegyptiacus*	*Eptesicus serotinus, Taphozous perforatus*	*Homo sapiens*

**Table 3:** List of bat species (Chiroptera) and their role as primary and secondary soft tick (Argasidae) hosts in the Western Palearctic (*N*, number of hosts with ticks).

**Bat species**	***N***	**Primary soft tick species**	**Secondary soft tick species**
*Asellia tridens*	3	*Chiropterargas boueti, Chiropterargas confusus*	*Carios vespertilionis*
*Barbastella barbastellus*	6	–	*Carios vespertilionis*
*Eptesicus isabellinus*	8	*Secretargas transgariepinus*	*Carios vespertilionis*
*Eptesicus nilssoni*	13	–	*Carios vespertilionis*
*Eptesicus serotinus*	54	*Secretargas transgariepinus, Carios vespertilionis*	*Reticulinasus salahi*
*Hypsugo savii*	14	*Secretargas transgariepinus*	*Carios vespertilionis*
*Miniopterus pallidus*	1	–	*Carios vespertilionis*
*Miniopterus schreibersii*	3	–	*Carios vespertilionis*
*Myotis alcathoe*	4	–	*Carios vespertilionis*
*Myotis bechsteinii*	1	–	*Carios vespertilionis*
*Myotis blythii*	1	–	*Carios vespertilionis*
*Myotis brandtii*	11	–	*Carios vespertilionis*
*Myotis dasycneme*	17	–	*Carios vespertilionis*
*Myotis daubentonii*	3	–	*Carios vespertilionis*
*Myotis emarginatus*	4	–	*Carios vespertilionis*
*Myotis myotis*	12	–	*Carios vespertilionis*
*Myotis mystacinus*	34	*Carios vespertilionis*	–
*Myotis nattereri*	7	–	*Carios vespertilionis*
*Nyctalus lasiopterus*	4	–	*Carios vespertilionis*
*Nyctalus leisleri*	14	–	*Carios vespertilionis*
*Nyctalus noctula*	47	*Carios vespertilionis*	–
*Nycteris thebaica*	2	*Chiropterargas boueti, Chiropterargas confusus*	–
*Otonycteris hemprichii*	5	*Chiropterargas boueti, Chiropterargas confusus*	*Secretargas transgariepinus, Carios vespertilionis*
*Pipistrellus kuhlii*	34	*Chiropterargas boueti, Chiropterargas confusus, Carios vespertilionis*	–
*Pipistrellus maderensis*	8	–	*Carios vespertilionis*
*Pipistrellus nathusii*	52	*Carios vespertilionis*	–
*Pipistrellus pipistrellus*	297	*Carios vespertilionis*	–
*Pipistrellus pygmaeus*	26	–	*Carios vespertilionis*
*Plecotus auritus*	24	–	*Carios vespertilionis*
*Plecotus austriacus*	21	*Secretargas transgariepinus*	*Carios vespertilionis*
*Plecotus christii*	3	–	*Secretargas transgariepinus, Carios vespertilionis*
*Plecotus gaisleri*	2	–	*Carios vespertilionis*
*Rhinolophus clivosus*	1	*Chiropterargas boueti*	*Carios vespertilionis*
*Rhinolophus ferrumequinum*	7	*Chiropterargas confusus*	*Carios vespertilionis*
*Rhinolophus mehelyi*	2	*Chiropterargas boueti*	*Carios vespertilionis*
*Rhinopoma cystops*	6	*Chiropterargas boueti, Chiropterargas confusus*	–
*Rhinopoma microphyllum*	1	*Chiropterargas boueti*	–
*Rousettus aegyptiacus*	11	*Chiropterargas boueti, Reticulinasus salahi*	*Carios vespertilionis*
*Tadarida aegyptiaca*	3	*Chiropterargas boueti, Chiropterargas confusus*	*Carios vespertilionis*
*Tadarida teniotis*	3	*Chiropterargas boueti*	*Carios vespertilionis*
*Taphozous nudiventris*	7	*Chiropterargas boueti, Chiropterargas confusus*	*Carios vespertilionis*
*Taphozous perforatus*	3	*Chiropterargas boueti, Chiropterargas confusus*	*Reticulinasus salahi*
*Vespertilio murinus*	56	*Carios vespertilionis*	–

Most tick records refer to subadult stages (only larvae being recorded on hosts, 93.13% of all ticks collected), with adults (males *n* = 25, females *n* = 67) and nymphs (*n* = 221) being collected from the environment or known bat roosts. Significantly more *Ca. vespertilionis* (mean intensity: 5.99 CI: 1.9–18.3) were collected from members of the genus *Pipistrellus* than from any other host species (*x*^2^ = 21.0216, *p* < 0.001).

**Figure 1:**
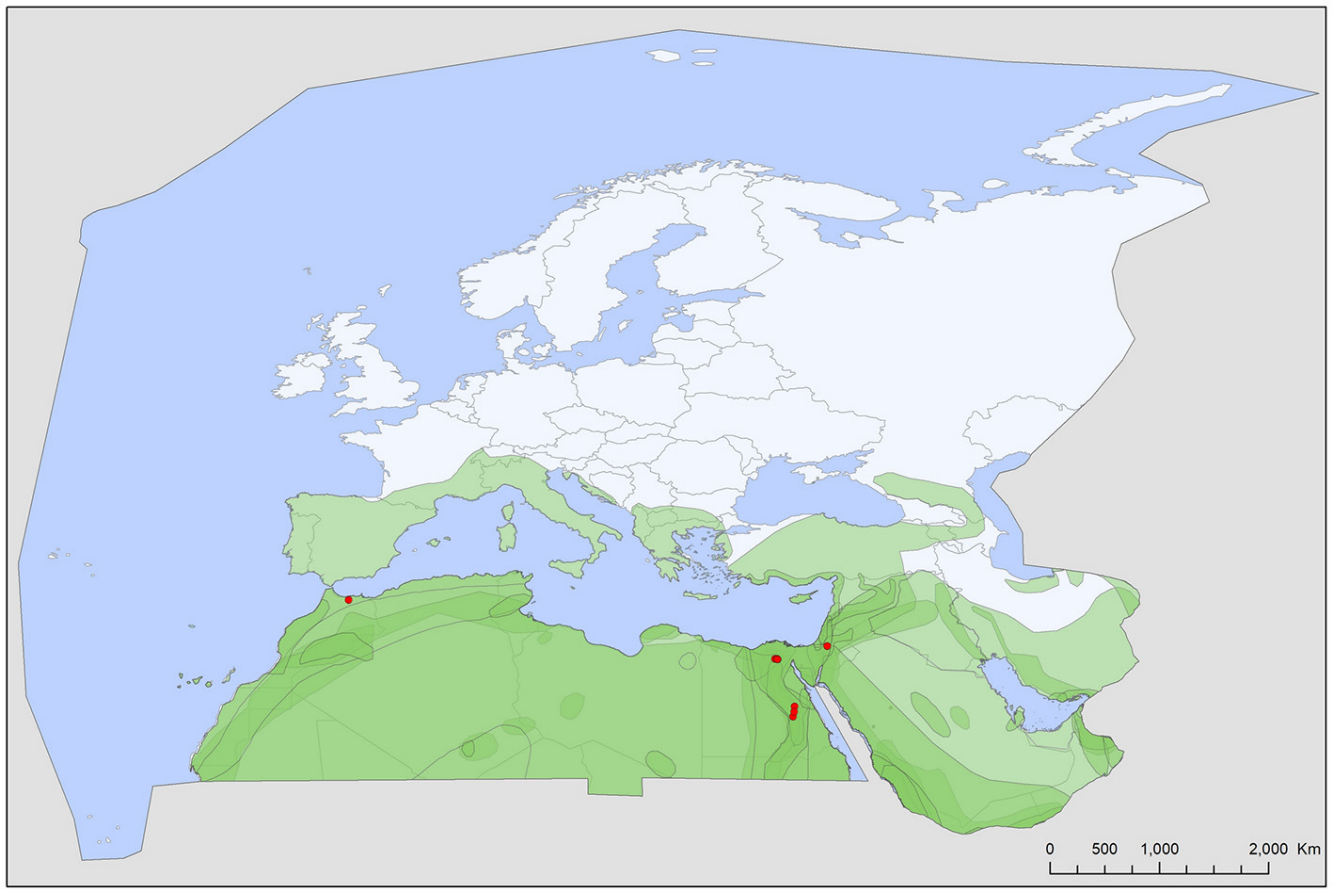
Geographic distribution of *Chiropterargas boueti* records in the Western Palearctic, overlayed to the geographic ranges for the 14 bat species studied as main hosts (Table 2) of this tick. Transparent layers were mapped on top of one another to highlight regions with dense range overlap. Some species have additional range overlap in Africa and Central and South Asia.

**Figure 2:**
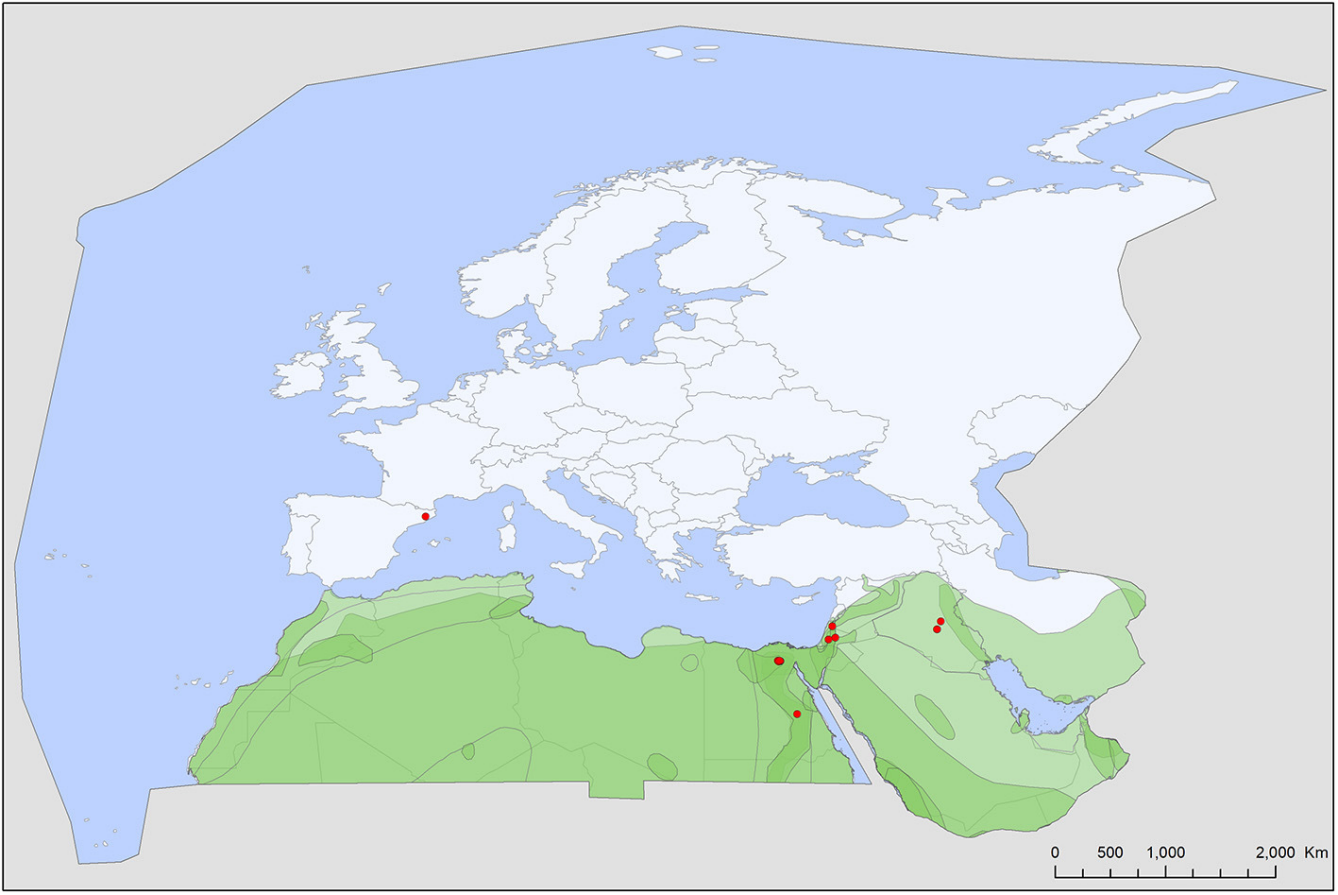
Geographic distribution of *Chiropterargas confusus* records in the Western Palearctic, overlayed to the geographic ranges for the nine bat species studied as main hosts (Table 2) of this tick. Transparent layers were mapped on top of one another to highlight regions with dense range overlap. Some species have additional range overlap in Africa and Central and South Asia.

**Figure 3:**
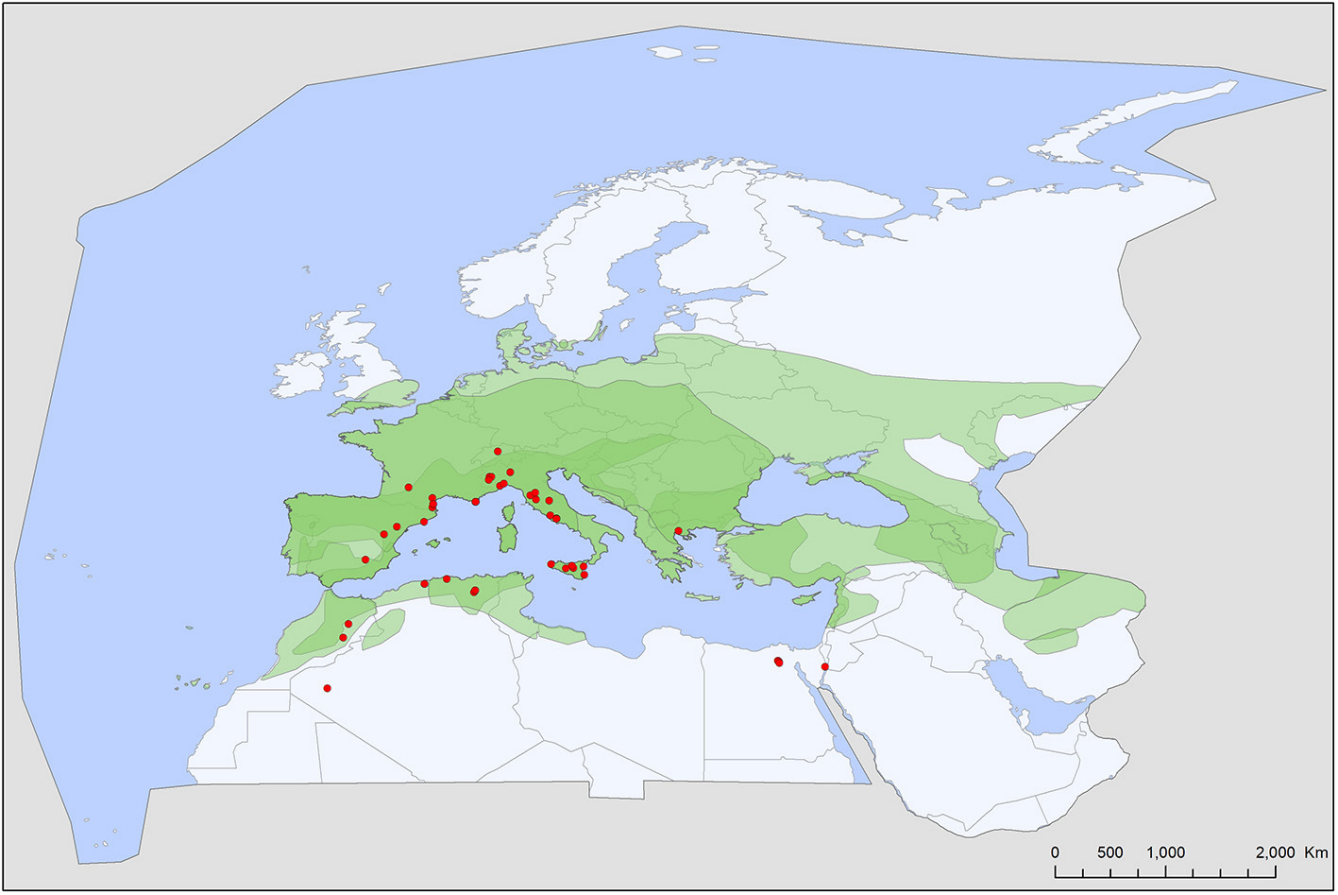
Geographic distribution of *Secretargas transgariepinus* records in the Western Palearctic, overlayed to the geographic ranges for the four bat species studied as main hosts (Table 2) of this tick. Transparent layers were mapped on top of one another to highlight regions with dense range overlap. Some species have additional range overlap in Africa and Central and South Asia.

**Figure 4:**
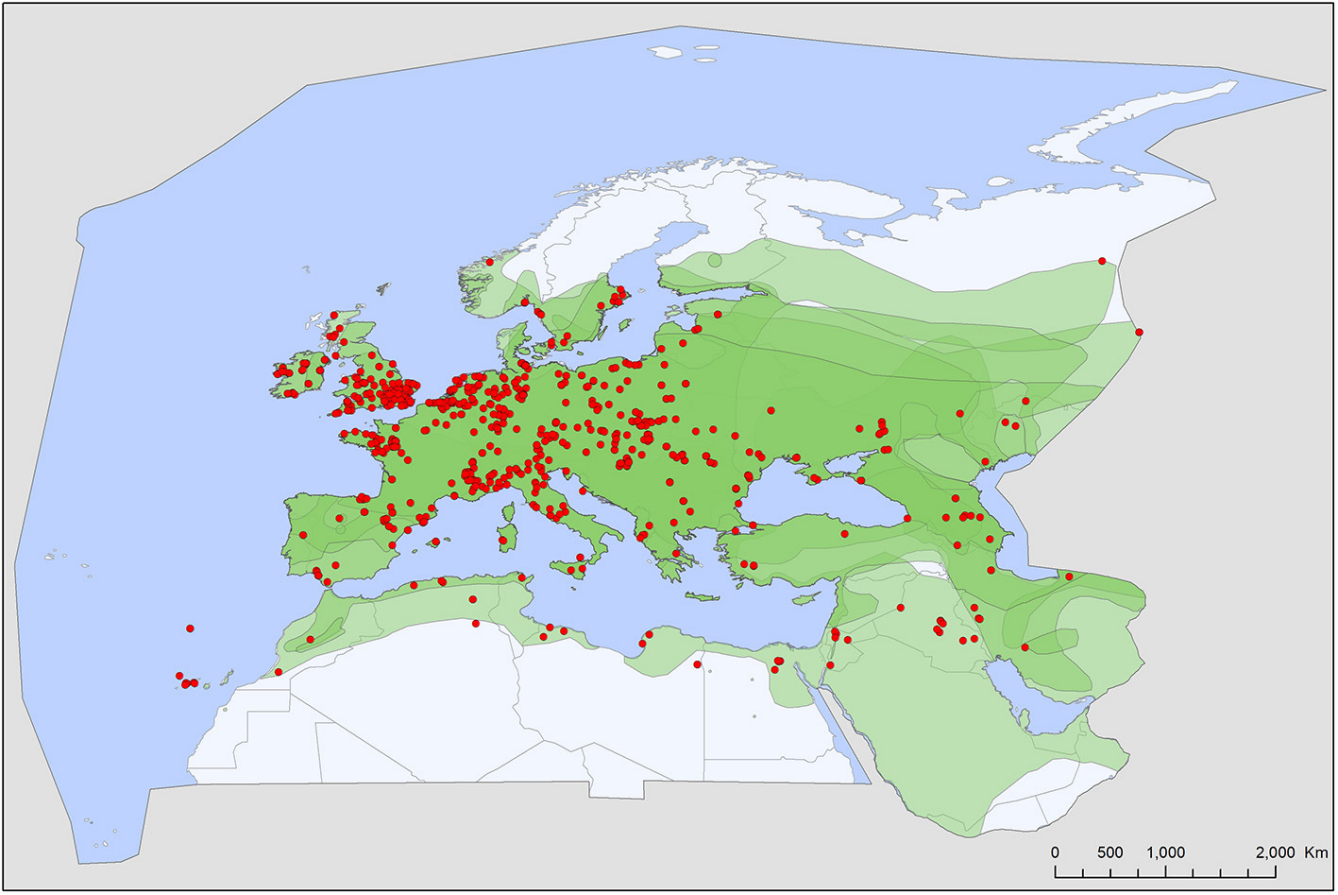
Geographic distribution of *Carios vespertilionis* records in the Western Palearctic, overlayed to the geographic ranges for the seven bat species studied as main hosts (Table 2) of this tick. Transparent layers were mapped on top of one another to highlight regions with dense range overlap. Some species have additional range overlap in Africa and Central and South Asia.

**Figure 5:**
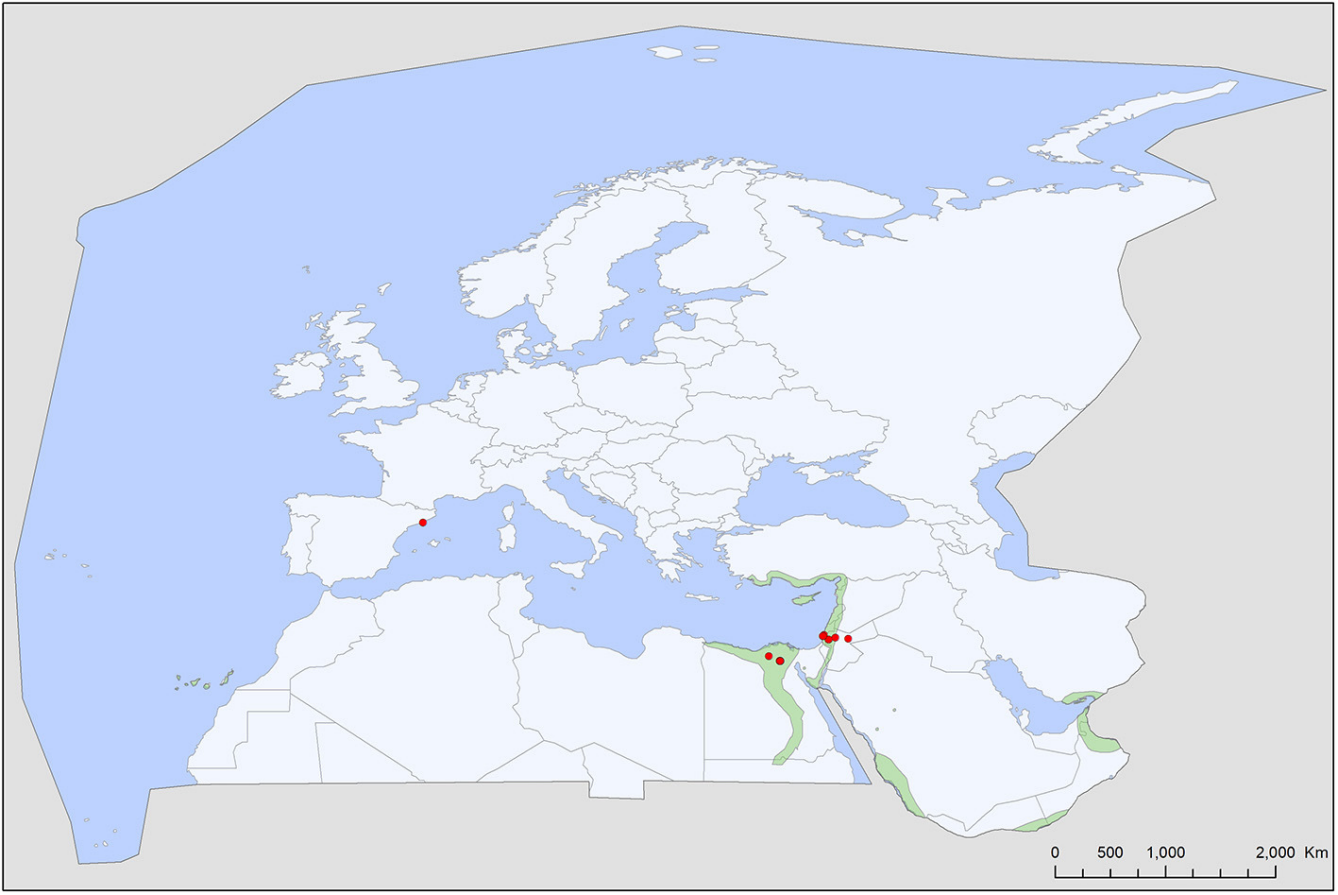
Geographic distribution of *Reticulinasus salahi* records in the Western Palearctic, overlayed to the geographic ranges for of *Rousettus aegyptiacus*, the sole primary host of this tick. *R. aegyptiacus* range extends into to the tropical and subtropical regions of Africa and Asia.

Soft tick records showed a wide geographic distribution, covering most of the Western Palearctic, with significant differences between the extents of individual ranges. All five soft tick species show overlapping ranges in North Africa, most species (4/5) had a primarily Mediterranean range, with *Ch. boueti, Chiropteraragas confusus*, and *R. salahi* being exclusively found in North Africa and the Middle East (Figures 1, 2, 5). *Carios vespertilionis* and *S. transgariepinus* are distributed also in Europe (Figures 3, 4). Most records of soft ticks came from bats caught in (or in immediate vicinity of) man-made structures (buildings, ruins, and underground channels: 66%), with 13.6% being collected from caves. The rest were collected from bats caught in diverse habitats (roost unknown) while hosts were in active flight.

Several viral, bacterial, and piroplasmid pathogens were identified in two soft tick species of bats. The most common groups were bacteria (*Bartonella* spp., *Borrelia* spp., *Coxiella burnetii*, and *Rickettsia* spp.), but also five different viruses (belonging to flaviviruses and nairoviruses), as well two piroplasmids (*Babesia* spp.) were identified in soft ticks of bats (Table 4).

**Table 4:** List of bacterial, protozoan, and viral pathogens identified in bat specialist soft ticks.

**Pathogen group**	**Pathogen species**	**Reference**
***Secretargas transgariepinus***		
Bacteria	*Rickettsia hoogstraalii*	(25, 26)
Viruses	Keterah (KTRO, nairoviruses)	(27)
***Carios vespertilionis***		
Bacteria	*Coxiella burnetii*	(28, 29)
	*Ehrlichia* sp. Av	(30)
	*Ehrlichia* sp. AvBat	(31)
	*Rickettsia africae*-like	(25)
	*Rickettsia helvetica*	(25)
	*Rickettsia lusitaniae*	(25)
	*Rickettsia raoultii*	(32)
	*Rickettsia rickettsii*	(32)
	*Rickettsia* sp. Av22	(25)
	*Rickettsia* sp. AvBat	(31)
	*Rickettsia* spp. (SFG group)	(30)
	*Bartonella* sp. Ia23	(25)
	*Bartonella* sp. Iv76	(25)
	*Bartonella* spp.	(19)
	*Borrelia afzelli*	(33)
	*Borrelia burgdorferi* s.l.	(34)
	*Borrelia* spp.	(35)
	*Borrelia* sp. CPB1 (“Relapsing Fever Group”)	(31)
	*Borrelia* spp. (“Relapsing Fever Group”)	(33)
Piroplasmida	*Babesia vesperuginis*	(30, 36–38)
	*Babesia venatorum*	(30)
Viruses	Issyk-Kul virus (IKV, nairoviruses)	(39–41)
	Keterah (KTRO, nairoviruses)	(27)
	Soft tick bunyavirus (STBV, nairoviruses)	(42)
	Sokuluk (SOKV, flaviviruses)	(41)
	Tick-borne encephalitis virus, (TBEV, flaviviruses)	(43)

## Discussion

**Figure 6:**
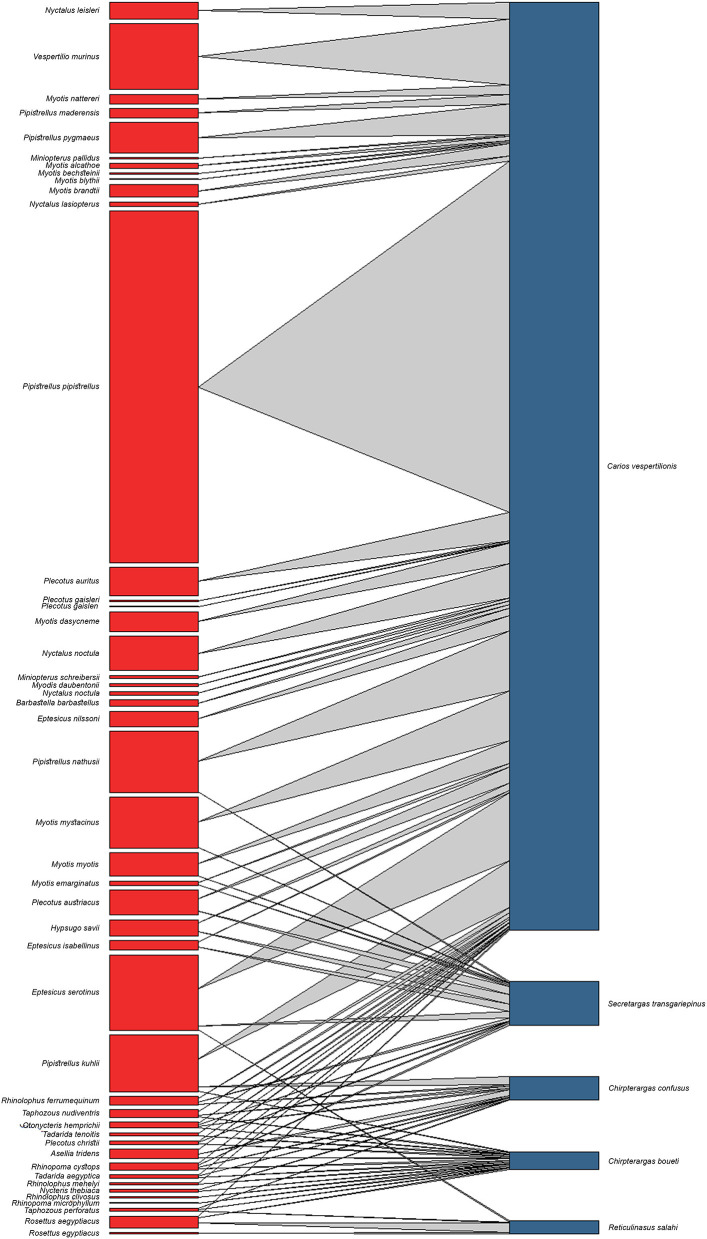
Quantitative interaction web based on bat specialist Argasidae ticks and their respective bat hosts. Links between nodes represent the sum of individual tick occurrences for a given bat species.

A total of five different soft tick species (Acari: Argasidae: *Ca. vespertilionis, Ch. boueti, Ch. confusus, R. salahi*, and *S. transgariepinus*) were recorded to be specialized to bats of the Western Palearctic. These ticks were found on 44 different bat species, showing diverse host-pattern (Figure 6). Most records came from a single tick species (*Ca. vespertilionis*, 88.7% of all records, Table 1), which not only has the highest number of host species, but also the widest distribution, covering the whole region of the Western Palearctic (Figure 4). Argasid ticks of bats primarily parasitize crevice dwelling host species, although there are three tick species (*Ch. boueti, Chiropteraragas confusus*, and *R. salahi*), for which most of the primary hosts are cave-dwelling bats. Soft tick occurrences showed a wide geographical distribution, covering most of the Western Palearctic. However, significant differences were found between the extent of individual ranges, with the range of three species being limited to North Africa and the Middle East. While overlapping areas are small, there is a region (northeastern part of Egypt and Israel) where all five species occur (Figures 1–5).

There is a considerable overlap between primary hosts among the different soft tick species. One bat species (*Pipistrellus kuhlii*) is the primary host for three different tick species, while further nine bat species regularly harbor two different argasid species (Table 2; Figure 6). Most tick species show a distribution that considerably overlaps with the range of their primary bat host (Figures 1, 2, 4, 5), with two notable exceptions. *Secretargas transgariepinus* shows a reduced range in comparison to its primary hosts' range, with several records in NE Africa, where primary hosts registered in the Western Palearctic do not occur (Figure 3). Records in this area came from bats exclusively distributed in Africa (*Rhinopoma* spp., *Taphozous* spp.), suggesting that on the African continent other primary hosts may occur. This species is well-known to regularly occur on bats performing large scale migrations like *Pipistrellus* spp. (24), hence several northern records may suggest accidental overshoots of argasid larvae collected from a bat in active migration (44). Another notable exception is the sole record of *R. salahi* in the Iberian Peninsula (Figure 5), far from the main range of its sole primary host, *Rousettus aegyptius*.

### Specific Accounts

*Chiropterargas boueti* is a very poorly known species. Most information on this species was published in the original description (45), as well in its redescription (46). It has a wide distribution, primarily on the African continent, reaching Central and South Africa, with scattered records in Central Asia and the Middle East (47, 48). It is primarily a tick of cave dwelling tropical bats, with primary host species being *Rhinopoma* spp., with an extralimital occurrence in the Western Palearctic (Figure 1). Its ecology and vectorial capacity is unknown, while it is known to attack humans (46).

*Chiropterargas confusus* has a similar occurrence to the previous species, with which it shares most of its primary host species and also the occurrence records in the Western Palearctic (Figure 2). Its ecology and distribution are poorly known, with only a handful of records listed in Northern, Eastern and Southern Africa and Central Asia (46, 48, 49). In the Western Palearctic, this species has a narrow range, with records in NE Africa and the Middle East. There is no published information on its vectorial role. There is a putative record of its occurrence on a non-bat host (50), suggesting its suitability as a more generalist tick species.

**Figure 7:**
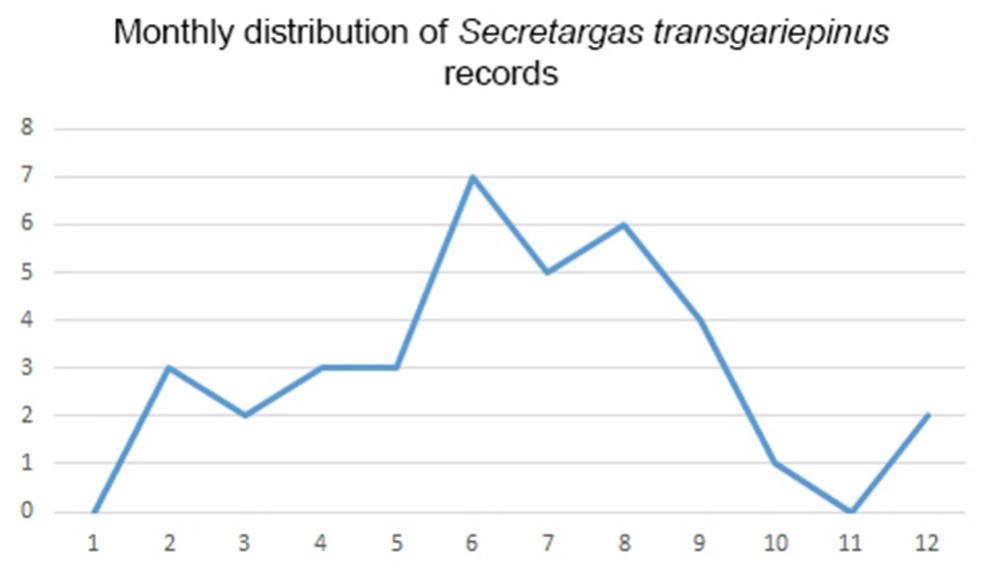
Monthly distribution of collection dates for *Secretargas transgariepinus* ticks.

*Secretargas transgariepinus* has a primarily tropical African distribution, with scattered records in North Africa and the Mediterranean region of Europe (51). It is primarily a parasite of crevice-dwelling bats, commonly occurring on *Eptesicus* spp., *Hypsugo savii*, and *Plecotus* spp. in the region (Tables 2, 3). The distribution of this argasid tick shows limited overlap with the range of its primary bat hosts in Europe, probably because its occurrence is limited by climatic factors (Figure 3). There is no clear seasonality in its records (Figure 7), and the apparent peak activity likely reflects an observation bias. The species is known for maternal care (52) and is a suspected vector (Table 4) for the Keterah virus (KTRO, nairoviruses) and spotted fever-causing bacteria of the genus *Rickettsia* (25–27). The species is regularly recorded on humans, with several cases known from Egypt and Italy (53).

**Figure 8:**
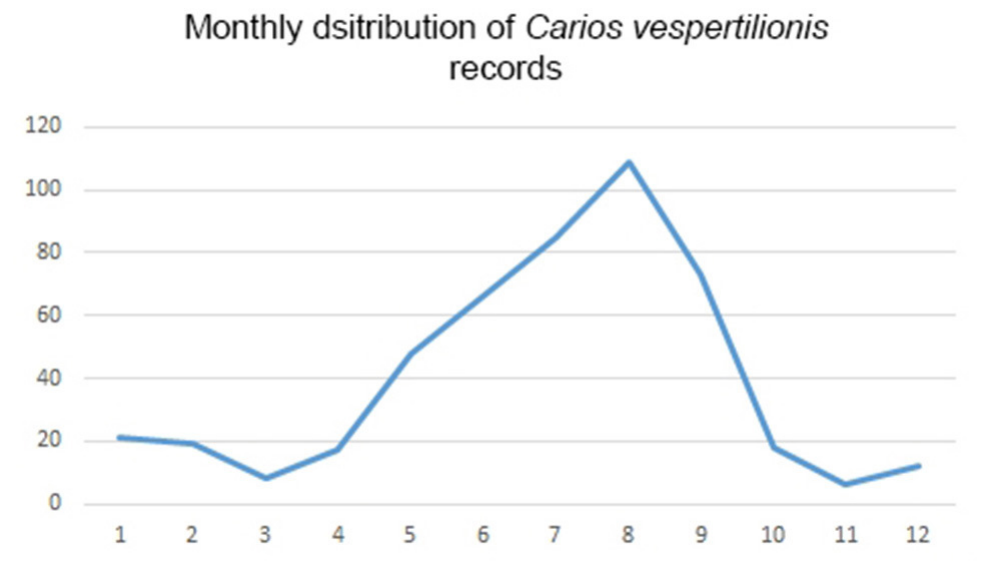
Monthly distribution of collection dates for *Carios vespertilionis* ticks.

*Carios vespertilionis* is the most common soft tick species of bats in the Western Palearctic (Table 1; Figure 4). It has the largest geographic distribution among bat ticks worldwide (54), with extensive morphological and genetic diversity along its wide range (36, 55). Its distribution mirrors the geographic range of the primary host species and it is the only soft tick species which may occur at the northernmost latitudes, wherever bats are present (Figure 4). It is also the species which has the highest number of records and known host species (Tables 1, 2). The species primarily occurs on crevice dwelling species (26 out of the 42 recorded host species, Table 2), with a particular affinity toward *Pipistrellus* spp., members of which usually host high number of individual ticks. These ticks may exert behavioral or even pathological impacts on their hosts (56), especially if they occur in high numbers (57, 58). While only larvae were recorded on hosts, roost sites (especially artificial ones) are important locations for adults, too (56, 59). This species was recorded in each month (Figure 8), and the seasonal distribution of records shows a summer peak. However, we suggest that this is mainly related to the timing of bat-research efforts in the field, rather than to a true activity peak of the ticks. *Carios vespertilionis* was recorded in multiple instances on humans (53, 60) and also on other vertebrates (Table 2) (61, 62). This species is known vector of several bacterial, protozoan and viral pathogens (Table 4 and references therein).

*Reticulinasus salahi* is the host specialist tick of the Egyptian fruit bat, *Rousettus aegyptiacus* (63). It occurs in the Western Palearctic only where its primary host is present (north-east corner of Africa and the Middle East, but missing from Cyprus, Figure 5). It's single European record came from an accidental host (64). There is no information published on its vectorial capacity, although several cases are known when humans were infested by this argasid species (53, 63).

Apart of the species listed above, a few accidental records refer to several other Palearctic soft tick species that may also accidentally infest bats, as exemplified by two bird-specialists (*Argas reflexus* and *Ornithodoros coniceps*) and a rodent specialist (*Ornithodoros tholozani*) (65–68).

Two out of the five bat-specialist soft ticks recorded in the Western Palearctic have a wide range. These species (*Ca. vespertilionis* and *S. transgariepinus*) are parasites of crevice-dwelling species in the Western Palearctic and both have a wide palette of primary and accidental host species (Table 1; Figure 6). Their host species are small to middle sized insectivorous bats, which do not depend on the accessibility of large underground roost sites and regularly roost is small groups, actively seeking anthropogenic shelters (24). As these bat species (chiefly *Pipistrellus* spp., the group of small *Myotis, Nyctalus noctula, Plecotus* spp. and *Eptesicus* spp.) are feeding mainly on flying small moths and dipterans (24), they easily can find food and shelter even in the most urbanized areas of the region. Hence, it is not a surprise that these species show increase both in their range and populations. In addition, they are among the few bat species which became true urban dwellers (69). Especially large urban settings offer to these species not only hunting areas (70) and roost sites in the active period, but also suitable hibernating areas. During the last decades it has become an increasing trend for several such bat species to use large buildings (e.g., multistorey office buildings and block of flats) for autumn congregations or wintering sites in major cities (71). This tendency increased not only the number of these bats inside highly urbanized areas (72), but also the contacts with humans (73, 74). These bat species regularly harbor soft ticks (while their roosts offer habitat for adult ticks), and both *Ca. vespertilionis* and *S. transgariepinus* are known to be competent vectors for a series of viral, bacterial and protozoan pathogens (Table 4), some of which are zoonotic. While *S. transgariepinus* is currently a rare species in the Western Palearctic, whose range is seemingly limited by climatic factors, increasing temperatures in the near future may favor further range extension for this species, especially as its hosts will possibly have broader distribution. If these trends will continue in the near future, the increasing presence of bats and their soft ticks may pose a new epidemiologic challenge in highly urbanized areas.

## Data Availability Statement

The original contributions presented in the study are included in the article/Supplementary Material, further inquiries can be directed to the corresponding author/s.

## Author Contributions

AS, AM, and SH designed the study and acquired the budget. AS and ÁP screened the reference publications and built the database. CD analyzed the data and created the maps. AS wrote the manuscript. All authors performed critical revision and approved the final manuscript.

## Conflict of Interest

The authors declare that the research was conducted in the absence of any commercial or financial relationships that could be construed as a potential conflict of interest.
